# Systemic multiple thrombosis caused by double homozygous mutations of PAI-1 4G/4G and MTHFR C677T during pregnancy

**DOI:** 10.1007/s44313-026-00124-7

**Published:** 2026-02-19

**Authors:** Hongcen Pan, Jinmi Li, Wenjun Zhu, Ruoxu Li, Jinan Jiang, Qing Huang

**Affiliations:** Department of Laboratory Medicine, Daping Hospital, Army Medical University, Chongqing, 400042 China

**To the editor,**

Pregnancy induces a physiological hypercoagulable state that significantly increases the risk of venous thromboembolism (VTE). Approximately 40% of affected pregnant women have hereditary thrombophilia factors [1]. The Plasminogen Activator Inhibitor-1 (PAI-1) 4G/4G homozygous mutation can inhibit fibrinolysis, and the Methylenetetrahydrofolate Reductase (MTHFR) C677T homozygous mutation may lead to hyperhomocysteinemia, both of which contribute to an elevated thrombotic risk. However, cases of extensive thrombosis resulting from the coexistence of these two mutations are rarely reported.

We report a case of a pregnant woman with a 4G/4G homozygous mutation in the PAI-1 gene and a C677T homozygous missense mutation in the MTHFR gene who subsequently developed multiple systemic VTE. The patient exhibited thrombosis in several vessels, including the portal vein, superior mesenteric vein, splenic vein, pulmonary artery branches, jugular vein, facial vein, and superficial veins of the upper limbs. Intestinal necrosis was subsequently observed. A multidisciplinary cooperation and treatment was implemented. It comprised portal vein incision thrombectomy, necrotic small bowel resection, portal vein catheter thrombolysis, step-by-step upgraded systemic anticoagulation (initially, intravenous infusion of heparin sodium, followed by subcutaneous injection of enoxaparin and oral administration of rivaroxaban), termination of pregnancy (artificial abortion via vacuum aspiration + curettage), and anti-infective measures. As a result, the patient’s condition was stabilized, the thrombus was effectively controlled, and the coagulation function returned to normal. This case indicates that in cases of unexplained widespread multiple thromboses during pregnancy, clinicians should maintain a high index of suspicion for hereditary thrombophilia, particularly the coexistence of PAI-1 and MTHFR gene mutations, which may substantially increase the risk of thrombosis.

A 24-year-old woman at 8 weeks of gestation was transferred to our hospital with persistent abdominal pain and vomiting that progressed despite treatment for suspected gastroenteritis and appendicitis. Before transfer, computed tomography imaging revealed extensive thrombosis in the portal and superior mesenteric veins.

The patient presented with an acute abdomen and bloody ascites, prompting immediate thrombectomy, small bowel resection, and portal vein catheterization. Owing to subsequent gastrointestinal bleeding, a cautious antithrombotic regimen was initiated. This regimen included portal vein catheter-directed recombinant human urokinase and intravenous heparin titrated to activated partial thromboplastin time and activated clotting time targets with concurrent supportive care.

The patient was assessed to be at high risk for VTE (Caprini score: 6) amid severe infection and a hypercoagulable state. External genetic testing confirmed double homozygous mutations in PAI-1 4G/4G and MTHFR C677T. Nevertheless, despite escalated heparin therapy (25.7 U/kg/h) and local thrombolysis, she developed recurrent thrombotic events, including cerebral infarction, pulmonary embolism, and venous thrombosis of the head, neck, and upper limbs, with D-dimer persistently elevated above 10,000 μg/L FEU (Table 1).

**Table 1:** Timeline of thrombotic events and key interventions

Hospital day	New thrombosis site and vent	Concurrent management
Day 0	Portal vein, superior mesenteric vein	Portal vein thrombectomy and intestinal resection surgery
Day 1	Portal vein, superior mesenteric vein, splenic vein, the tip of the internal jugular vein catheter	Catheter-directed thrombolysis initiated
Day 3	Right frontal lobe cerebral infarction, thrombosis in the branch of the left lower pulmonary artery	Systemic heparin sodium escalated
Day 5	Right facial vein, left jugular vein	
Day 8	Left cephalic vein	
Day 10		Decision and procedure for pregnancy termination
Day 15	Right radial vein	
Day 20	PICC site in the right axillary vein, right cephalic vein	
Day 23	Remove the central venous catheter	Portal vein catheter removed; heparin discontinued
Days 33 and 34	Right basilic vein, left internal jugular vein	
Day 45	Decrease in platelet count	Switch to rivaroxaban

*PICC* peripherally inserted central catheter

A pivotal decision to terminate the pregnancy was made on hospital day 10 because of her critical condition and the refractory nature of the thrombosis, despite maximal anticoagulation. Heparin was withheld for 2 h pre-procedure and resumed post-procedure at escalated doses (17.1–21.4 U/kg/h). Following curettage, the serum levels of human chorionic gonadotropin and D-dimer decreased markedly (Figs. 1 and 2), improving the state of high coagulation. Consequently, on hospital day 23, the portal vein catheter was removed, and heparin was discontinued.

**Fig. 1:**
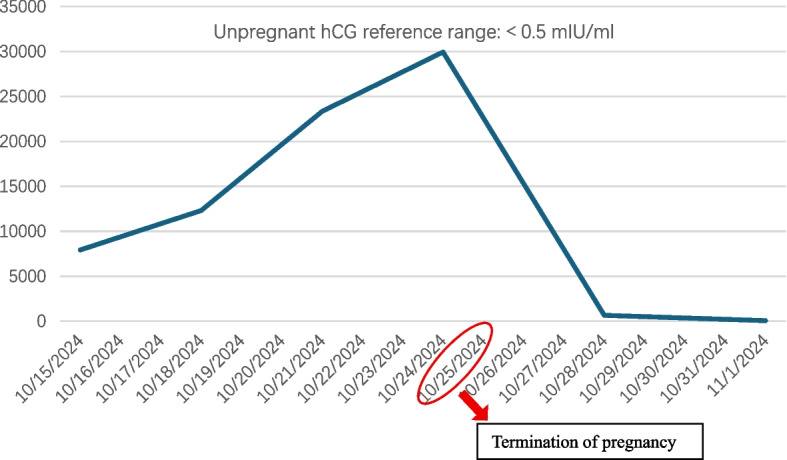
Trend of serum hCG levels during hospitalization. hCG: human chorionic gonadotropin

**Fig. 2:**
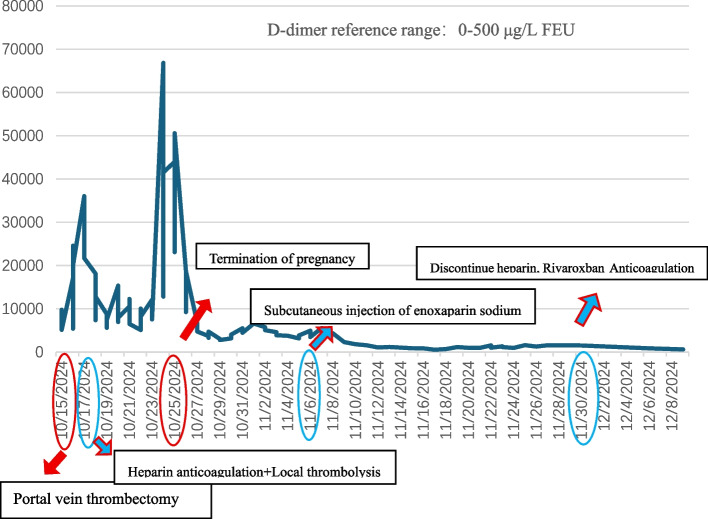
Trend of plasma D-dimer levels during hospitalization

After catheter removal, the anticoagulation therapy was switched to enoxaparin sodium. However, the platelet count gradually declined. Considering the patient’s medication history, heparin-induced thrombocytopenia was strongly suspected. Given the high thrombus burden and clinical risk, the clinical decision was to empirically discontinue all heparin-based medications. After switching to rivaroxaban, the patient’s coagulation parameters and D-dimer levels normalized. The patient was discharged on rivaroxaban, and the coagulation parameters remained stable during the 6-month follow-up period.

This case report describes a rare presentation of extensive thrombosis involving the portal venous system, pulmonary arteries, and multiple superficial veins. Widespread thrombosis in this patient likely resulted from an interplay between hereditary and acquired factors; the hypercoagulable state of pregnancy served as the central driver, while surgery and infection further promoted coagulation. Furthermore, the coexistence of the MTHFR C677T and PAI-1 4G/4G polymorphisms may have produced a synergistic effect, collectively exacerbating thrombotic risk.

During pregnancy, enhanced coagulation factor activity, decreased anticoagulant levels, and suppressed fibrinolysis lead to a hypercoagulable state [2]. This disruption of the coagulation-anticoagulation-fibrinolysis balance significantly increases the risk of VTE in pregnant women [1].

PAI-1 plays a key role in maintaining hemostatic balance by inhibiting the activity of tissue- and urokinase-type plasminogen activators, thereby reducing fibrin degradation [3]. Studies have confirmed that, compared with the PAI-1 4G/5G and 5G/5G genotypes, the PAI-1 4G/4G genotype is associated with higher plasma PAI-1 activity and is an independent risk factor for VTE [4]. Notably, PAI-1 activity significantly increases during pregnancy [5]. Additionally, pathological conditions such as surgical trauma or infection can stimulate endothelial cells to synthesize more PAI-1 by promoting the release of inflammatory cytokines [6].

The MTHFR C677T mutation results in reduced enzyme activity, leading to hyperhomocysteinemia and an elevated risk of VTE [7]. Some studies have suggested a notable association between the MTHFR C677T polymorphism and the risk of thrombosis [8], whereas others have suggested that its capacity to increase the risk of thrombosis is restricted unless accompanied by hyperhomocysteinemia [9]. Therefore, the precise prothrombotic contribution of the homozygous MTHFR C677T mutation in this case remains unclear. The combination of the severe hypercoagulable state of pregnancy, surgical stress, and systemic infection may have amplified its effect.

Nevertheless, Wang et al. indicated that the synergistic effect of homozygous MTHFR and PAI-1 gene mutations significantly increased VTE risk [10], exceeding the sum of the risks associated with each mutation alone [11]. Pasta et al. also suggested that PAI-1 4G/4G and MTHFR 677TT polymorphisms enhance inflammatory responses and activate hepatic stellate cells, leading to liver fibrosis and increased intrahepatic vascular resistance, elevating portal pressure. Both play crucial roles in the formation of portal vein thrombosis [12]. Given that this patient developed widespread multicentric thrombosis within a short period, the synergistic effect between hereditary and acquired factors cannot be overlooked.

The management of this case was challenging. A multidisciplinary team was also established. Key treatments included portal vein thrombectomy, resection of necrotic small bowel, portal vein catheter-directed thrombolysis, systemic anticoagulation transitioning to oral rivaroxaban, uterine evacuation, and anti-infective therapy. The patient’s condition eventually stabilized. Following pregnancy termination, the patient’s estrogen levels decreased, accompanied by a significant reduction in D-dimer levels. Moreover, anti-infective and anticoagulation treatments were consistently administered and were associated with notable alleviation of the patient’s hypercoagulable state.

In conclusion, this case demonstrates a synergy between hereditary genetic mutations and the procoagulant challenges posed by pregnancy. It highlights the importance of suspecting complex hereditary thrombophilia in cases of unexplained extensive thrombosis, considering pregnancy termination in refractory cases, and relying on multidisciplinary care for comprehensive management.

## Data Availability

No datasets were generated or analysed during the current study.
